# Archetype Analysis of Lung Adenocarcinoma Premalignancy Links Heterogeneity in Premalignant Lesions to Diverging Features of Invasive Disease

**DOI:** 10.1158/1541-7786.MCR-25-1093

**Published:** 2026-05-06

**Authors:** Kelley E. Anderson, Linh M. Tran, Kostyantyn Krysan, Yohana Kefella, Lingge Yu, Gregory A. Fishbein, Erika F. Rodriguez, Maryam Shabihkhani, Daniel P. Stefanko, Emily Green, Gang Liu, Hanqiao Liu, Sherry Zhang, Erin Kane, Mitra Mehrad, Avrum E. Spira, Steven M. Dubinett, Eric J. Burks, Sarah A. Mazzilli, Marc E. Lenburg, Jennifer E. Beane

**Affiliations:** 1 Section of Computational Biomedicine, Boston University Chobanian and Avedisian School of Medicine, Boston, Massachusetts.; 2 Department of Medicine, David Geffen School of Medicine at UCLA, Los Angeles, California.; 3 Department of Pathology and Laboratory Medicine, David Geffen School of Medicine at UCLA, Los Angeles, California.; 4 Department of Pathology, Microbiology, and Immunology, Vanderbilt University Medical Center, Nashville, Tennessee.; 5 Department of Pathology, Boston University Chobanian and Avedisian School of Medicine, Boston, Massachusetts.

## Abstract

Lung adenocarcinoma (LUAD), the most common subtype of lung cancer, is a heterogeneous disease with highly variable histopathologic features and clinical outcomes. LUAD premalignant lesions (PML) are localized proliferations of atypical pneumocytes that spread along the alveolar walls. In this study, we characterized the molecular heterogeneity across PMLs, regardless of histologic classification, and identified four distinct groups or “archetypes” of PMLs using bulk RNA and DNA exome sequencing data from laser-captured microdissected PMLs, normal, and tumor tissues from LUAD resection cases. The archetypes were defined byrecurrent gene coexpression modules discovered using the LUAD PMLs we profiled and three publicly available datasets. One PML archetype, termed “proliferation,” was associated with increased expression of genes involved in cell proliferation, a protumor immune environment, and enrichment for *EGFR* driver mutations. In contrast, the “normal-like” archetype exhibited molecular features similar to normal tissue, an antitumor immune response, and lacked driver mutation enrichment. We projected the PML archetypes into independent gene expression datasets profiling LUAD and found that tumors closest to the proliferation archetype were enriched for multiple features of aggressive LUAD, including high tumor grade and lymph node invasion, and were significantly associated with shorterdisease-free survival. Proliferation archetype PMLs may represent a subset of lesions with increased malignant potential and may provide opportunities to improve patient stratification and identify novel targets for lung cancer interception.

**Implications:**

Molecular signatures that distinguish indolent from aggressive LUAD PMLs may help personalize lung cancer screening and interception protocols.

## Introduction

Lung cancer is the most common cause of cancer-related death, both worldwide (1) and in the United States, with an overall 5-year relative survival of 26% (2). The most common form of lung cancer is lung adenocarcinoma (LUAD). LUAD has highly variable clinical outcomes and is heterogeneous with respect to histopathologic features and molecular alterations. LUAD premalignant lesions (PML) are precursors to LUAD and include atypical adenomatous hyperplasia (AAH, a localized proliferation of atypical type II pneumocytes ≤ 5 mm), adenocarcinoma *in situ* (AIS, proliferation with pure lepidic growth ≤ 30 mm without invasion or necrosis), and minimally invasive adenocarcinoma (MIA, predominantly lepidic growth measuring ≤ 30 mm and with ≤ 5  mm of a nonlepidic component or stromal invasion; ref. 3). AAH and AIS/MIA typically appear as ground glass opacities (GGO) on lung computed tomography (CT) scans, and approximately 20% of these lesions will progress to invasive malignancy (4). With the expanding adoption of lung cancer screening programs, the detection of PMLs via lung CT is anticipated to rise substantially (5), and advanced robotic bronchoscopy techniques are beginning to provide an opportunity to biopsy these lesions. The clinical management of PMLs, however, remains a key challenge. Imaging and molecular biomarkers may be useful to accurately stratify the risk of lesion progression to invasive disease (6). Biomarkers capable of preoperatively identifying indolent preinvasive LUAD have the potential to mitigate overtreatment of screen-detected nodules (7), whereas biomarkers specific for aggressive PMLs will ensure appropriate intervention.

Prior work has identified molecular features associated with premalignant and early-stage LUAD histologic progression, including somatic mutations (8–13), transcriptional alterations (10, 11, 14–17), and altered immune responses (11–14, 16). However, recent evidence from a study of LUAD premalignancy in a cohort of East Asian never-smokers suggests that gene expression changes do not follow a linear progression from AAH to AIS to MIA during carcinogenesis (18), indicating that molecular changes among PMLs may not directly parallel histologic progression. Although many of the prior studies contained limited PML samples, they highlight the potential to enhance histopathologic grading of PMLs by integrating molecular features. Archetype analysis has been proposed as a framework for understanding tumor heterogeneity (19, 20) and has been used to discover new targets against small cell lung cancer’s aggressive features (21). To better understand heterogeneity among LUAD PMLs, we defined archetypes through analysis of bulk RNA sequencing of PMLs collected from a retrospective cohort of patients and public datasets. Archetyping identified heterogeneity in gene expression patterns and pathway activity among LUAD PMLs, independent of PML histology. Using whole-exome sequencing and multiplex immunofluorescence (mIF), we linked the archetypes to driver mutations and features of genomic instability, as well as to altered density of immune cell types and proliferating epithelial cells. Projection of the archetypes into independent LUAD gene expression datasets demonstrated their association with LUAD histopathologic features and disease-free survival. Characterizing PML archetypes may enable identification of aggressive PMLs prior to their progression to invasive LUAD, as well as identify indolent PMLs that can be monitored via imaging surveillance.

## Materials and Methods

### Study population and sample collection

**Figure 1.**
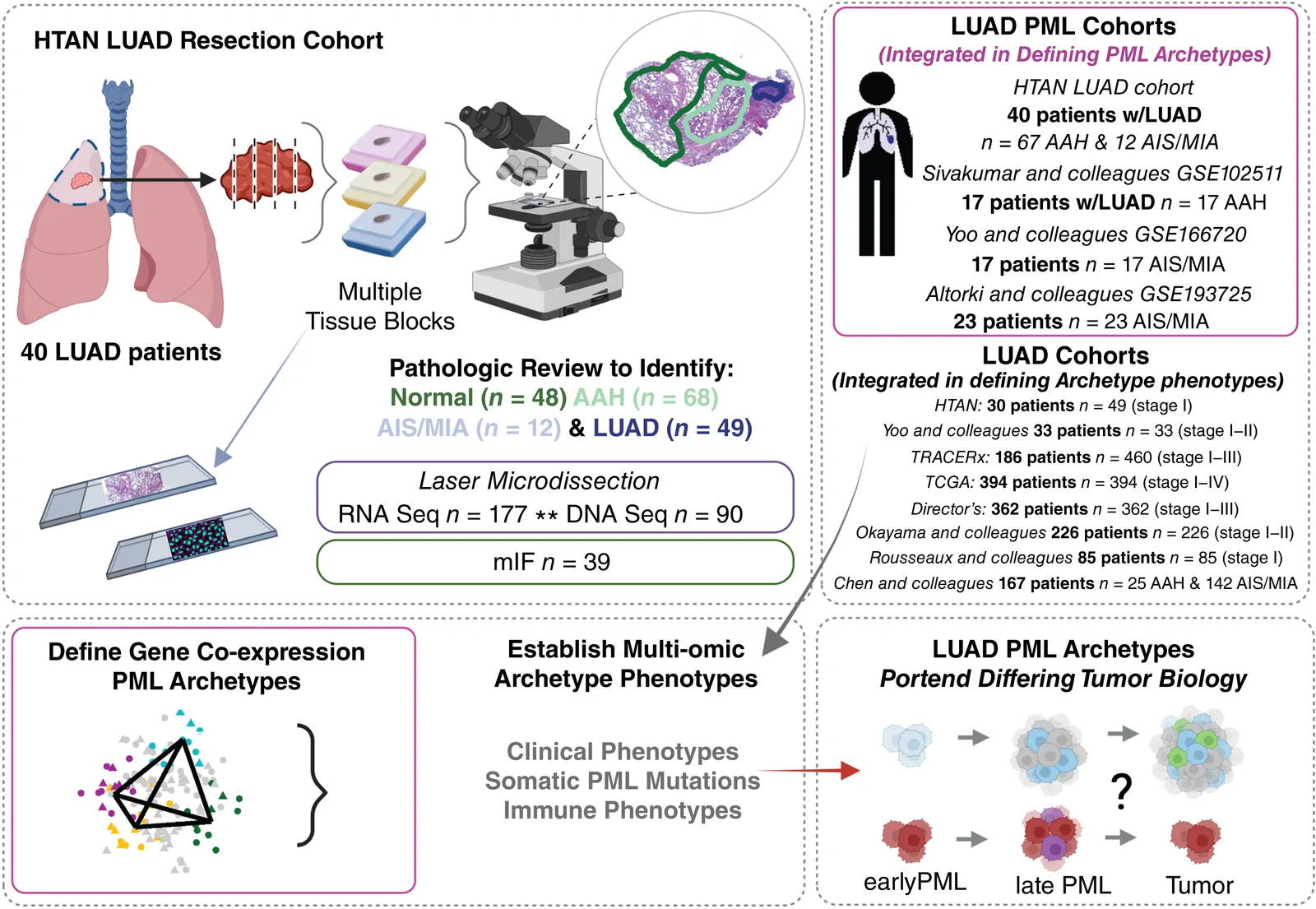
The HTAN Lung PCA cohort and multiple publicly available datasets were used to identify LUAD PML archetypes and to associate these archetypes with clinical phenotypes of LUAD tumors. Participants in the Lung PCA cohort (*n* = 40 patients) underwent resection for LUAD. Normal, PML, and tumor tissues from the resection were profiled by bulk RNA-seq (*n* = 177 samples), with subsets of these samples additionally profiled by whole-exome sequencing (*n* = 90 samples) and/or mIF (*n* = 39 samples). The Lung PCA RNA-seq data, together with three additional PML cohorts, were used to define four molecular archetypes. The archetypes were then projected into large-scale LUAD studies to evaluate associations between PML molecular features and clinical and histopathologic features of indolent vs. aggressive LUAD. [Figure was created in BioRender. Mazzilli, S. (2026) https://BioRender.com/y253r09.]

Patients that underwent resection for LUAD consented to the use of remanent tissue for research, including molecular profiling. Cases were reviewed by a clinical pathologist at each enrollment site to identify tumors with additional premalignant histology within the resected tissue as part of the Lung Pre-Cancer Atlas (Lung PCA) supported by the National Cancer Institute Consortium for Integrated Molecular, Cellular, and Imaging Characterization of Screen-Detected Lung Cancer and the Human Tumor Atlas Network (HTAN, RRID: SCR_023364). Formalin-fixed, paraffin-embedded (FFPE) tissue from each patient’s resection (lobectomy, wedge resection, or segmentectomy) was annotated to identify normal, tumor, and at least 1 area of premalignant histology for laser-capture microdissection (LCM; Supplementary File S1). Each PML sample is an independent lesion identified in the tumor resection tissue blocks (Fig. 1). We utilized LCM to isolate the normal, PML, and tumor histology in FFPE tissue sections to enrich for the cells of interest associated with each histology and minimize contamination with the surrounding tissue (*n* = 218 total samples). Tissue histology was reviewed by an independent pathologist, and any samples without agreement by two pathologists were removed from the analysis (*n* = 26 samples). RNA and DNA were isolated from the same LCM tissue to ensure high fidelity in genomic comparisons. Samples with sufficient RNA were profiled with bulk RNA sequencing (RNA-seq), and samples with high-quality sequencing data were selected for use in our analysis (*n* = 177 samples from *n* = 40 patients after quality filtering). Of these samples, any with sufficient DNA were profiled via whole-exome sequencing (*n* = 90 samples from *n* = 25 patients after quality filtering). Samples with sufficient tissue were also profiled using mIF staining (*n* = 39 samples). The high-quality data included patients from the University of California, Los Angeles (*n* = 119 normal, PML, and tumor samples, *n* = 24 patients), Vanderbilt University Medical Center (*n* = 30 normal, PML, and tumor samples, *n* = 9 patients), and Boston Medical Center (*n* = 28 normal and tumor samples, *n* = 7 patients).

### RNA-seq library preparation, sequencing, and data processing

RNA from LCM FFPE samples was isolated using the Qiagen All-Prep FFPE Kit (RRID: SCR_008539), and sequencing libraries were prepared using the Illumina TruSeq Paired-End Cluster Kit (RRID: SCR_010233) using samples with sufficient RNA quality and quantity (*n* = 6 samples discarded). Total RNA-seq was performed on an Illumina HiSeq 2500 instrument (RRID: SCR_016383) to generate paired-end 100-nucleotide reads. After sequencing, FASTQ files for each sample were aligned to *hg38* using *STAR* (RRID: SCR_005622; ref. 22), and *RSEM* (RRID: SCR_000262; ref. 23) was used to summarize gene and transcript counts. Quality metrics were generated by *STAR* and *RSeQC* (RRID: SCR_005275; ref. 24) and used to evaluate sample quality. *Somalier* (25) was used to confirm relatedness among samples from the same patient. *EdgeR 3.25.10* (RRID: SCR_012802; ref. 26) was used to compute normalized data by using the trimmed mean of M-values normalization and computing log_2_-counts per million (log-CPM) in *R 3.6.0* (RRID: SCR_001905).

Gene filtering was performed using the *filterByExpr* function in *edgeR* to keep genes that had approximately 0.6 counts per million or more in at least 48 samples, with the cutoff based on the median library size (17.7 million) and the histology variable (where there are 48 normal samples as the smallest group) to avoid giving preference to samples with larger library sizes. Samples were excluded (*n* = 9 samples discarded) if they had values greater than 2 standard deviations from the mean for one or more criteria: (i) the first or second principal components (PC) calculated using log-CPM across the filtered gene set, (ii) median transcript integrity number, or (iii) lack of relatedness among samples from the same patient as indicated by *Somalier* analysis. After sample filtering, gene filtering was recomputed as described above on the final set of high-quality samples (*n* = 177 samples, *n* = 21,218 genes). We used *ComBat-seq* (27) to adjust for batch effects (*n* = 2 batches) in the Lung PCA cohort. For each sample, we ranked genes to find the top 1,000 genes with the highest total number of counts and then calculated the number of counts coming from those top 1,000 genes. We divided this value by total counts for each sample to identify samples in which a disproportionate share of counts is concentrated in a small set of highly expressed genes. This metric is significantly correlated (*r* = −0.4, *P* < 0.0001) to the *RSeQC* rate of uniquely mapped reads in the Lung PCA cohort and was computed for publicly available data sets utilized in subsequent analyses.

### Exome DNA-seq library preparation, sequencing, and data processing

DNA from LCM FFPE samples was isolated using the Qiagen All-Prep FFPE Kit and sequencing libraries constructed utilizing the Roche KAPA Kapa Hyper Prep Kit (KK8514), followed by exome enrichment with the IDT xGen Exome Research Panel v2. DNA sequencing was performed on an Illumina NovaSeq 6000 (RRID: SCR_016387) instrument to generate paired-end 150-nucleotide reads. Genomic DNA from the paired normal lung tissue served as the germline control for each patient. There is no significant difference in age (*t* test, *P* = 0.69), sex (χ^2^, *P* = 0.62), tumor stage (χ^2^, *P* = 0.68), smoking status (χ^2^, *P* = 0.98), or tumor subtype (χ^2^, *P* = 0.93) between the subset of patients with both exome sequencing and RNA-seq data compared with samples with RNA-seq in the Lung PCA cohort. Somatic variants were identified using two different variant calling methods: *Mutect2* (bioRxiv 2019.12.02.861054; RRID: SCR_026692) and *Strelka2* (28). A mutation was called if it passed mutation calls by both algorithms. Variants in exon, splicing, and untranslated regions were assessed with a focus on exonic single-nucleotide variants (SNV). Driver mutations identified by The Cancer Genome Atlas (TCGA) in LUAD were assessed, as well as those previously identified in AAH and LUAD (10, 12, 29). Each sample’s mutant-allele tumor heterogeneity (MATH) score was computed as described by Mroz and Rocco (30): (i) the mutant-allele fraction for each locus was calculated as the ratio of mutant loci to total reads, (ii) the median absolute deviation was computed as the absolute difference between the mutant-allele fraction and the median mutant-allele fraction, (iii) the MATH score was computed as the ratio of the median absolute deviation to the median of the mutant-allele fractions among the tumor-mutated loci. Jaccard similarity was computed as the ratio of the intersection of the shared deletions, insertions, and single-nucleotide polymorphisms (SNP) to their union for the PML and tumor samples. *Maftools* (RRID: SCR_024519; ref. 31) was used to analyze and plot SNVs.

### Graph-based clustering to identify PML consensus gene modules

We sought to construct a set of gene modules designed to be robust across different LUAD PML–related data sets by adapting a method previously used to identify consensus modules that defined recurrent cancer cell states across different cancer types (32). For each of the PML sample groups from both the Lung PCA cohort (*n* = 68 AAH, *n* = 12 AIS/MIA) and the three additional datasets, sample and gene filtering were performed as described above for each data set independently. Each of the three additional data sets were accessed from Gene Expression Omnibus (GEO; RRID: SCR_005012) using the *GEOquery 2.51.6* (RRID: SCR_000146; ref. 33) package to import the gene count matrices into R: GSE102511 (paired normal/PML/tumor samples from 17 patients, *n* = 17 AAH, *n* = 15 normal, and *n* = 15 LUAD; ref. 10), GSE166720 (independent samples from 50 patients with either early-stage LUAD or MIA/AIS, *n* = 17 AIS/MIA and *n* = 33 LUAD following filtering with two samples removed; ref. 15), and GSE193725 (paired normal/PML, normal/tumor, and independent samples from 42 total patients, *n* = 23 AIS/MIA, *n* = 10 normal, and *n* = 18 LUAD; ref. 17).

For each dataset, we constructed an adjacency matrix by computing Pearson correlations between gene pairs, setting negative coefficients to zero, and raising positive coefficients to the power of six. For each individual adjacency matrix, coefficients equal or greater than the 99th percentile were set to one, and the rest were set to zero. By using coefficient cutoffs based on percentiles computed for each data set, we attempted to avoid biasing the results toward data sets with higher or lower overall correlations due to technical factors. The binarized (0, 1) matrices were summed, so that gene pairs with a strong correlation in multiple datasets would have values greater than 1. The summed matrix was again binarized (0, 1) by setting gene–gene connections with a value of 3 or greater (the gene pair was connected in 3 or more datasets) to 1 and all other connections to 0. We then filtered out genes with few connections to other genes by removing genes with a row sum less than 10. Graph clustering performed using *infomap* clustering implemented in *igraph 1.3.5* (RRID: SCR_019225; ref. 34) resulted in a set of nine gene modules (*n* = 1628 genes). Gene Ontology (RRID: SCR_002811) Biological Process 2023 gene sets and mSigDB “Hallmark” gene sets (RRID: SCR_016863; ref. 35) enriched (false discovery rate, FDR < 0.05) among genes in each module were identified using *EnrichR* (RRID: SCR_001575; ref. 36) using *Ensembl* IDs converted to gene symbols with *biomaRt 2.39.4* (37).

### Identification of archetypes

We used PMLs from four datasets to define archetypes (Lung PCA, GSE102511, GSE166720, and GSE193725). The intersection of filtered genes from each dataset was used to create one gene by sample count matrix (*n* = 137 samples, *n* = 15,968 genes). We computed gene expression residual values for each sample using the *residuals* function in *limma 3.39.19* (RRID: SCR_010943; ref. 38), adjusting for study and the percentage of gene counts coming from the top 1,000 genes for each sample. The residual gene expression matrix was used in all downstream analyses. Gene set variation analysis (GSVA; RRID: SCR_021058; ref. 39) was used to compute a sample score for each of the nine PML-derived modules that was used as input to the archetype analysis conducted using *ParetoTI 0.1.13* (RRID: SCR_022991; refs. 40, 41) to find the positions of archetypes of a polytope (principal convex hull) that best described the data. The archetype analysis was adapted from methods used to identify archetypes in small cell lung cancer (21). We determined the explained variance for different numbers of archetypes, *k,* and chose the optimal number of archetypes, *k*^*#*^, by finding the “elbow” of the explained variance versus *k* curve. The elbow represents *k*^*#*^, where the explained variance starts decreasing in linear fashion when adding additional archetypes. The elbow was identified by finding the *k* most distant from the line that passes through the first (*k* = 2) and last (*k*_*max*_ = 10) points in the explained variance versus *k* graph. We confirmed *k*^*#*^ using the *t* ratio metric that measures similarity between the data and the computed polytope, with a higher *t* ratio indicating higher similarity. The *t* ratio *P* value for a given *k* (*k* = 3 to *k* = 6) was computed empirically as the number of times, out of 5,000, that a computed *t* ratio based on randomized gene modules exceeded the *t* ratio. The randomized gene modules were created by randomly choosing genes (from the *n* = 1628 gene set) to create nine modules with identical sizes to the original modules. The lowest *k* with a significant (*P* < 0.05) *t* ratio was chosen as the optimal number of archetypes.

Samples were scored based on their distance from each vertex (archetype center) of the polytope. We projected the data into PC space to visualize the relationship of the samples to the archetypes in 2D space, and for each archetype, a subset of samples was assigned to an archetype. The octile (0.125) of samples closest to each archetype were assigned as primary samples of that archetype. The 0.125 distance quantile was selected to maximize the number of samples with a single archetype assignment while keeping the fraction of samples with multiple assignments to less than 5%. After assigning samples to the archetypes, we confirmed that none of the four archetypes had categorically assigned samples that were significantly further or closer to the archetype center compared with each of the other archetypes (Wilcox test, *P* > 0.4 for all comparisons). To ensure that the selected cutoff (0.125) used for assigning archetypes to samples did not unduly influence the results, we confirmed the associations between archetypes and variables of interest using all samples and their continuous distance to each archetype vertex with linear modeling throughout our analysis. Distance from each archetype can be computed for every sample, regardless of archetype assignment.

### Characterization of archetypes

Each archetype was annotated based on (i) significant associations (FDR<0.05) between PML-derived gene module GSVA scores and distance from the archetype identified using linear mixed-effects models in which the module GSVA score and dataset were fixed effects and patient was a random effect, (ii) significant associations (FDR <0.05) between published gene signatures (10, 42) summarized per sample using GSVA scores and distance from the archetype identified using linear mixed-effects models in which the signature GSVA score and dataset were fixed effects and patient was a random effect, and (iii) significant enrichment (FDR <0.05) of mSigDB “Hallmark” gene sets (35) among genes with increased expression in samples closest to each archetype using gene set enrichment analysis (GSEA; RRID: SCR_003199) with a preranked gene list (43, 44). For GSEA, genes were ranked by *t*-statistic for their association with distance from each archetype based on linear mixed-effect models that included distance from the archetype, study, and the percentage of counts from the most highly expressed genes as fixed effects and patient as a random effect. All models were fit using *voom*-transformed (45) data and *limma* (38) with the “duplicateCorrelation” function to account for within-patient correlation. The FDR was used to adjust *P* values for linear models.

Linear models, linear mixed-effects models, and logistic mixed-effect models were used throughout to assess the associations between archetypes and variables of interest. In all analyses including samples from more than one study, study was included as a fixed effect. In analyses including samples from cohorts with more than one sample per patient (Lung PCA), patient was included as a random effect. When the variable of interest had more than two levels, binary indicators were created to compare one level versus the others (e.g., proliferation archetype PMLs vs. all other PMLs). For models that used the Lung PCA MATH scores, contrasts were used to compare between tumor samples and PML samples with assigned archetypes. A logistic mixed-effects model with distance from archetype and study as fixed effects and patient as a random effect was used to determine the association between distance from each archetype and the probability of harboring different driver mutations. Tumor immune dysfunction and exclusion (TIDE) scores were computed using an interface (46) developed to evaluate gene expression profiles of cancer samples to determine their potential for tumor immune escape, and a logistic mixed-effects model was used to determine the association between distance from each archetype and probability of benefiting versus not from immunotherapy, with distance and study as fixed effects and patient as a random effect. χ^2^ tests were used to determine the association between assigned archetype samples and clinical or pathologic variables (histology, dataset, smoking status, or sex). For each χ^2^ test contingency table, we computed an overall χ^2^ statistic and residuals for each cell in the table to determine which row and column combinations contributed the most to the total χ^2^ statistic. mIF staining and analysis was performed on samples, with cell density calculated based on the tissue area, as described and previously published by Yanagawa and colleagues (47).

### Projection of archetypes into PML and LUAD datasets

Archetype space is continuous, so transcriptome profiles from other data sets can be placed in the polytope that defines the archetypes (21). Additional microarray datasets [GSE68465 (48), GSE31210 (49, 50), and GSE30219 (51)] were accessed from GEO using *GEOquery* to import the data into R and then log_2_ transformed. For bulk RNA-seq data [TCGA (29), Tracking Cancer Evolution through Therapy (TRACERx; ref. 52), Lung PCA tumors, GSE166720, and Chen and colleagues (18)], sample and gene filtering as well as gene expression normalization were performed as described above for each dataset. TCGA (RRID: SCR_003193) data were downloaded from https://portal.gdc.cancer.gov. Chen and colleagues data were accessed from the European Genome-phenome Archive (RRID: SCR_004944) at accession number EGAD50000000637. TRACERx data were downloaded from https://doi.org/10.5281/zenodo.7683605 and https://doi.org/10.5281/zenodo.7603386. For each dataset, we computed GSVA scores for each of the nine PML-derived modules on normalized gene expression data and used these GSVA scores to compute the Euclidean distance of each sample to each of the stored locations of the PML-derived archetype centers. To visualize the samples relative to the archetype positions, we used *ParetoTI* (40, 41) to project the stored positions of the archetypes from our PML analysis into the PC space computed for each independent LUAD dataset.

We used several methods to determine the relationship between the archetypes and LUAD clinical and histopathologic features in each independent LUAD dataset and in LUAD samples from the Lung PCA and GSE166720 datasets. χ^2^ tests were used to determine the association between samples assigned archetypes and (i) sample histology in the Lung PCA data, (ii) tumor grade and lymph node involvement in the GSE68465 data, and (iii) sample histology and density on CT in the Chen and colleagues data. To determine the association between distance from each archetype center and LUAD predominant histologic subtype (lepidic, solid, etc.), linear mixed-effect models were used with a binary variable for tumor histologic pattern (e.g., solid tumor histology versus other histology) and stage as fixed effects and patient as a random effect where applicable (TRACERx and Lung PCA). To determine the association between distance from each archetype center and lymph node spread, linear mixed-effects models were used with a binary variable for no lymph node disease (N0) versus N1 or above and stage as fixed effects and patient as a random effect in datasets with multiple samples per patient. For stage I tumors in each LUAD dataset, the hazard ratio (HR) was estimated using a Cox proportional-hazard model of disease-free survival as a function of categorical archetype or archetype distance, with patient included as a random effect in the TRACERx dataset that contained multiple samples per patient.

## Results

### Study population

**Figure 2.**
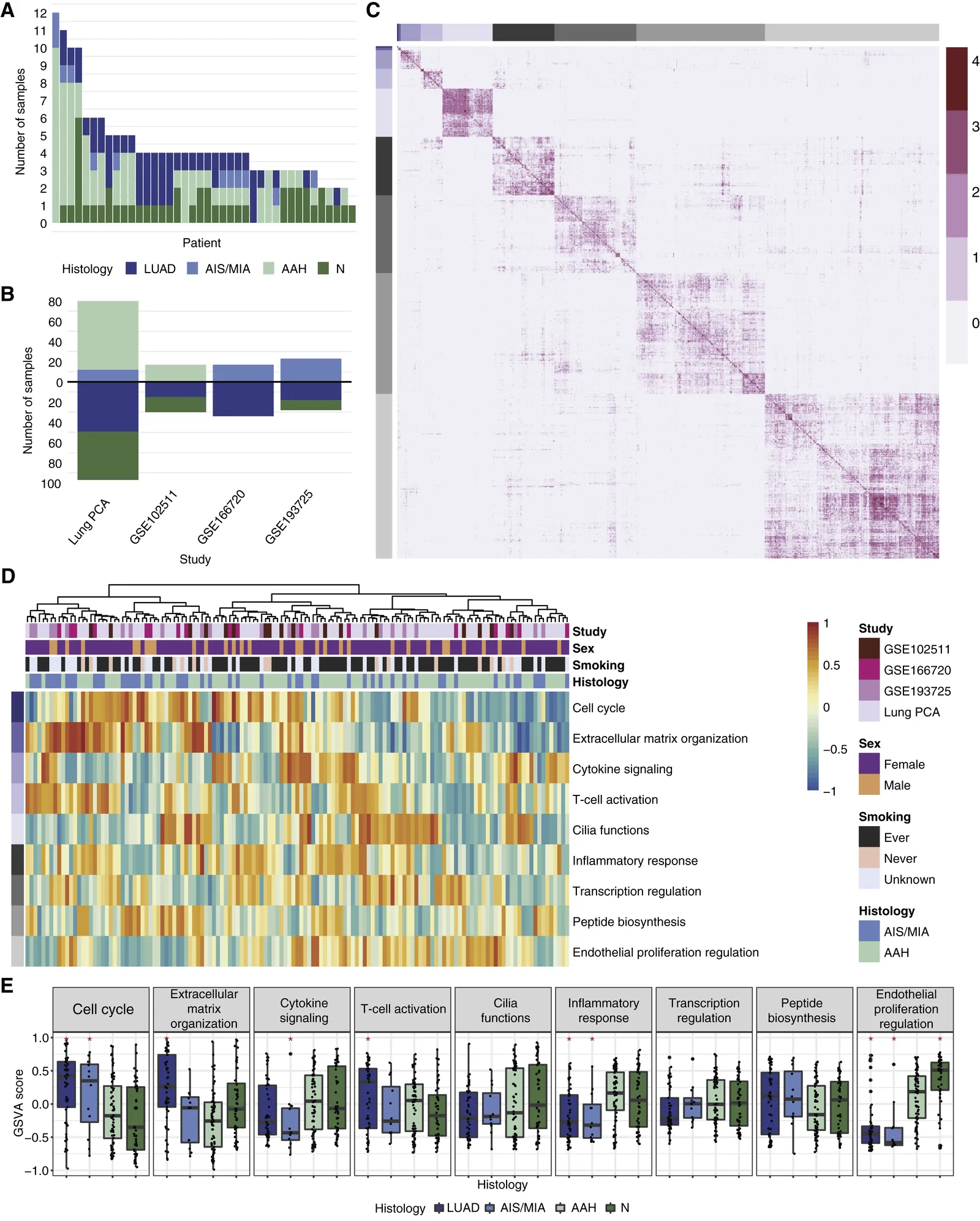
Gene modules recur across studies of LUAD premalignancy. **A,** Stacked bar plot of sample histology per patient from the Lung PCA cohort (*n* = 40 patients, *n* = 177 samples following filtering). **B,** Stacked bar plot showing the histology distribution for each cohort utilized in this analysis. The cohorts included were the Lung PCA (*n* = 80 PML, *n* = 48 normal samples, and *n* = 49 tumor samples), GSE102511 which contained paired normal/PML/tumor samples from 17 patients (*n* = 17 PML, *n* = 15 normal samples, and *n* = 15 tumor samples), GSE166720 which contained independent samples from 50 patients with either early-stage LUAD or MIA/AIS (*n* = 17 PML and *n* = 33 tumor samples), and GSE193725 which contained a mixture of paired normal/PML, normal/tumor, and independent samples from 42 total patients (*n* = 23 PML, *n* = 10 normal samples, and *n* = 18 tumor samples). **C,** Heatmap of summed binarized gene adjacency matrices from each of the four PML datasets after filtering to identify highly connected genes (*n* = 1628 genes). Heatmap shades indicate number of datasets in which the genes are correlated, ranging from 0 (white) to 4 (deep red). Rows/columns are semi-supervised by modules identified using *igraph* clustering, with color annotations indicating each module. **D,** Heatmap of GSVA scores computed from each PML-derived gene module. Row labels indicate pathway enrichment of the nine modules, and row colors correspond to modules identified in **C**. Columns are hierarchically clustered using *ward.d2* similarity of the Euclidean distance of samples. **E,** Boxplots of GSVA scores computed for each PML-derived module across the Lung PCA cohort samples (*n* = 177 samples) stratified by module and colored by histology. *, histology is significantly different from AAH samples (linear mixed-effects models with histology as a fixed effect and patient as a random effect, *P* < 0.05).

Tissue blocks were obtained from a retrospective cohort of patients (Fig. 1), collected as part of the HTAN Lung PCA, that underwent surgical LUAD resection at Boston Medical Center, Vanderbilt University Medical Center, and University of California, Los Angeles Health (Table 1). RNA and DNA were isolated from LCM of PML, tumor, and normal tissues from fixed tissue sections from the surgical resection of each patient. RNA was subjected to bulk RNA-seq (Supplementary File S1; Fig. 2A, *n* = 177 total samples after filtering from *n* = 40 total patients, with *n* = 68 AAH and *n* = 12 AIS/MIA samples from *n* = 29 patients). Samples were predominantly early-stage LUAD (80% stage I) from older patients (mean age 68.7 ± 9.6 years) with a history of tobacco use (62.5% former smokers). A representative subset (see Materials and Methods) of these tissue samples underwent exome sequencing (*n* = 90 LUAD and PMLs from *n* = 25 patients), using paired normal samples from each patient as the respective germline control.

**Table 1.** Phenotypic and clinical features of lung PCA cohort patients.

Variable	Patient (*n* = 40)
Site of tissue collection	​
Boston Medical Center	7 (17.5%)
University of California, Los Angeles Health	24 (60%)
Vanderbilt University Medical Center	9 (22.5%)
Smoking status	​
Current	10 (25%)
Former	25 (62.5%)
Never	5 (12.5%)
Sex	​
Female	30 (75%)
Male	10 (25%)
Age at diagnosis	68.7 ± 9.6
Predominant histologic pattern	​
AIS	3 (7.5%)
MIA	3 (7.5%)
Lepidic	4 (10%)
Acinar	10 (25%)
Papillary	4 (10%)
Micropapillary	1 (2.5%)
Solid	3 (7.5%)
Invasive mucinous	3 (7.5%)
Not specified	9 (22.5%)
Pathologic stage	​
0	3 (7.5%)
I	32 (80%)
II	2 (5%)
IIIA	2 (5%)
Unknown	1 (2.5%)
Race	​
Asian Indian or Pakistani, NOS	1 (2.5%)
Black	2 (5%)
Vietnamese	1 (2.5%)
Other Asian, including Asian NOS	1 (2.5%)
White	24 (60%)
Unknown	7 (17.5%)

NOTE: Patient numbers and percentages are reported for categorical variables. Mean and standard deviation are reported for continuous variables.

Abbreviation: NOS, not otherwise specified.

We also used bulk RNA-seq data from LUAD PML samples from three previously published datasets. Sivakumar and colleagues (GSE102511; ref. 10) performed RNA and targeted DNA sequencing to identify transcriptional changes and driver mutations from a cohort of East Asian patients diagnosed with predominantly stage I LUAD (RNA-seq samples included *n* = 17 AAH from *n* = 17 patients). This cohort was similar to the Lung PCA study design and provides increased patient diversity from a geocultural perspective. Yoo and colleagues (GSE166720; ref. 15) and Altorki and colleagues (GSE193725; ref. 17) performed RNA-seq on patients who had only AIS/MIA (*n* = 17 AIS/MIA from *n* = 17 patients and *n* = 23 AIS/MIA from *n* = 23 patients, respectively) or stage I/II LUAD (Fig. 2B). We selected these two studies to increase the number of AIS/MIA samples in our analysis and to include PMLs in patients without LUAD tumors because field cancerization (53) may affect gene expression profiles of tissue adjacent to tumors.

### Identification of gene coexpression modules in LUAD premalignancy

To identify novel patterns of transcriptional heterogeneity among LUAD PMLs, we used a discovery-based approach to identify PML archetypes based on patterns of gene module coexpression. To accomplish this, we first derived a set of gene modules that consisted of genes with robust coexpression across multiple LUAD PML data sets (Fig. 2B). To identify recurring gene coexpression relationships, we identified gene–gene pairs with strong expression correlation in at least three datasets and further selected genes that demonstrated connections to multiple other genes, resulting in a total of 1628 genes (Fig. 2C). The filtered gene connection matrix was clustered using *igraph* (34), and nine gene modules were identified (average number of genes = 180, minimum = 5, maximum = 523). We observed, for each module and dataset, that genes within a module were significantly more correlated with the module GSVA (39) score than to genes outside the module (Supplementary Fig. S1A, Kolmogorov–Smirnov test, *P* < 0.0001), confirming consistent coexpression of the modules across data sets. Module genes were significantly enriched (FDR < 0.05) for “Biological Process” Gene Ontology terms via *EnrichR* (36) and included diverse processes such as extracellular matrix organization, cell cycle, and cytokine signaling (Supplementary File S2, Supplementary File S3). Module GSVA scores across all samples were orthogonal (average module-module GSVA score Pearson R = 0.12 ± 0.097) in accordance with their unique biology (Fig. 2D). To quantify the relationship of individual module GSVA scores with histology, using AAH samples as the basis for comparison, we found that the cell cycle and extracellular matrix organization modules were higher in tumors compared with AAH, with cell cycle also being higher in AIS/MIA compared with AAH. In contrast, the regulation of endothelial proliferation module was increased in normal samples compared with AAH (Fig. 2E). These associations were also preserved in the three additional datasets used to derive the modules (Supplementary Fig. S1B, S1C, and S1D). Across datasets, the gene modules had unique conserved patterns of coexpression and biology and demonstrated significant associations with histology.

### Gene coexpression modules identify four distinct archetypes of PMLs

**Figure 3.**
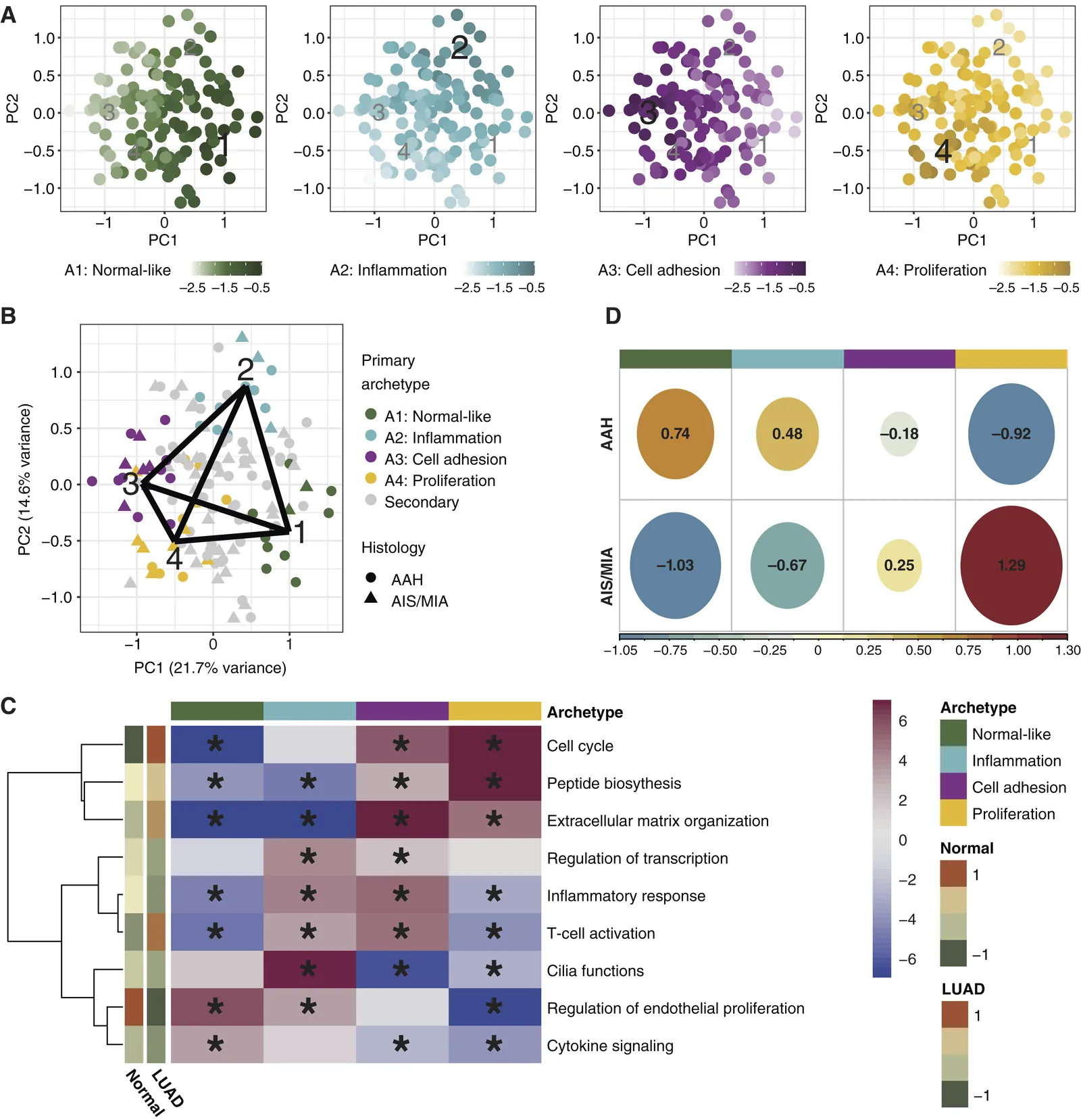
Archetype analysis on PMLs demonstrates correlation of archetypes with molecular and clinical phenotypes. **A,** Scatterplots of first two PCs computed from GSVA scores for PML-derived modules across PML samples from all four cohorts. Each plot is colored by the distance of each sample from an archetype (vertex of polytope). Each sample has four associated distances—one for each archetype vertex. More negative numbers reflect samples further away from the vertex. **B,** Scatterplot of first two PCs computed from GSVA scores for PML-derived modules for all PML samples. Samples in the top 0.125 proportion of distances from each archetype were assigned archetypes, whereas other samples were labeled as “secondary,” with colors representing samples closest to each respective archetype. Shapes indicate histology of samples. **C,** Heatmap of *t*-statistics from linear mixed-effects models of GSVA scores for each PML-derived module (rows) modeled with distance from each archetype (columns) and study as fixed effects and patient as a random effect.*, FDR < 0.05 for the distance term. Rows are hierarchically clustered using *ward.d2* similarity of the Euclidean distance. Row annotations represent the median GSVA score for each module in Lung PCA samples of either normal tissue or tumor. **D,** Visualization of χ^2^ residuals from χ^2^ test of association between archetypes and histology. Plot shows contribution of each comparison in the matrix to the test statistic, where circle size is proportional to contribution. Positive (red) values specify a positive association between the corresponding row and column variables (χ^2^ test, *P* = 0.18, *n* = 37 AAH and *n* = 19 AIS/MIA).

We hypothesized that the module coexpression heterogeneity among PMLs might be driven by different molecular phenotypes. We therefore sought to use the PML-derived gene modules to define archetypes (19, 40) of LUAD PMLs, an approach previously used to identify phenotypic heterogeneity in small cell lung cancer (21). To avoid biasing the results toward modules with larger numbers of genes, we used the GSVA scores for each module (Fig. 2D) as the input and included all PML samples used to identify the gene modules. We determined that four archetypes best fit the data based on a graph of explained variance versus *k* (Supplementary Fig. S2A; Materials and Methods) and a *t* ratio (54) permutation test (Supplementary Fig. S2B; Materials and Methods). Samples were scored based on their distance from each vertex of the polytope (archetype center) and visualized in PC space using PC1 and PC2 (Fig. 3A). The octiles of samples (12.5%), with the highest similarity scores to each archetype, were assigned to that archetype (Fig. 3B; Materials and Methods). If samples had overlapping primary assignments (*n* = 6 samples, 4.4% of samples) or did not have assignments (*n* = 75), we labeled them as “secondary” (*n* = 81 total, 59.1% of samples). We designated the four archetypes as follows: normal-like (A1, green, *n* = 12 samples, 8.8% of samples), inflammation (A2, blue, *n* = 13 samples, 9.5% of samples), cell adhesion (A3, purple, *n* = 16 samples, 11.7% of samples), and proliferation (A4, yellow, *n* = 15 samples, 10.9% of samples).

Next, we characterized the archetypes using the premise that samples close to an archetype share specific biological functions (19, 40). We identified PML-derived gene module scores that were significantly associated with distance from each archetype (Fig. 3C, FDR<0.05 indicated with asterisk) and Molecular Signatures Database (MSigDB) “Hallmark” gene signatures (35, 55) that were significantly enriched among genes correlated with archetype distances using GSEA (Supplementary Fig. S3, only pathways with positive normalized enrichment score and FDR <0.05 are shown; refs. 43, 44). Samples close to the normal-like archetype were positively associated with increased GSVA scores for the regulation of endothelial cell proliferation module (FDR <0.0001), which was also significantly higher in samples with normal histology compared with AAH lesions (Fig. 2E, *P* = 0.0011). The normal-like archetype was also associated with pathways that may regulate cell proliferation (P53) or activate apoptotic pathways (UV response upregulation). Samples closer to both the inflammation and cell adhesion archetypes had significantly higher GSVA scores for the T-cell activation (FDR <0.0001) and inflammatory response (FDR <0.0001) modules and upregulation of Hallmark immune-related pathways (Supplementary Fig. S3). The extracellular matrix organization module expression was a marked difference between these archetypes as samples close to the cell adhesion and inflammation archetypes were positively and negatively associated (FDR <0.0001) with the module, respectively. Samples close to the cell adhesion archetype also had increased expression of Hallmark angiogenesis, epithelial-to-mesenchymal transition, and *Wnt* and hedgehog signaling pathways, suggesting a molecular phenotype linked to cell migration. Samples close to the proliferation archetype were most positively associated with increased GSVA scores for the cell-cycle module (FDR <0.0001), which was also significantly higher in tumor samples compared with AAH samples (Fig. 2E, *P* < 0.0001). Proliferation archetype samples also had increased expression of genes in pathways associated with cell proliferation, metabolic functions, and oxidative stress in the Hallmark gene set.

We also determined the associations between clinical and histopathologic variables and the archetypes using samples with primary archetype assignments and across all samples using each sample’s continuous distance to each archetype vertex. The proliferation archetype was the only archetype significantly associated with AIS/MIA versus AAH histology (*n* = 85 AAH and *n* = 52 AIS/MIA, logistic regression, *P* = 0.021). Among samples with an archetype assignment, AIS/MIA samples were positively enriched, but not statistically significantly, in the proliferation archetype samples compared with other archetypes (Fig. 3D; Supplementary Table S1, χ^2^, *P* = 0.18, *n* = 37 AAH and *n* = 19 AIS/MIA). There were no significant associations between archetype assignments and dataset, smoking status, or sex (Supplementary Fig. S4A–S4C, χ^2^). We also tested whether the distance from each archetype was associated with datasets in which PMLs were primarily sampled near LUAD tumors (Lung PCA, Sivakumar and colleagues/GSE102511) and datasets in which PMLs were sampled in the absence of tumors (Altorki and colleagues/GSE193725, Yoo and colleagues/GSE166720). We observed no significant association between distance from each archetype and whether PMLs were primarily sampled in proximity to a tumor or not (logistic regression, *P* > 0.05 for all archetypes).

### Archetypes are associated with the somatic mutation landscape in premalignancy

To identify the relationship between LUAD PML archetypes and somatic mutations, we first examined MATH (30) across samples in the Lung PCA cohort with exome sequencing data (Supplementary File S1, *n* = 26 LUAD, *n* = 12 AIS/MIA, and *n* = 52 AAH samples from *n* = 25 patients). MATH is a measure of the mutant-allele fraction, in which heterogenous tumors with many genetically distinct subclones will have a higher MATH score. Tumor samples had a significantly higher MATH score compared with PMLs (linear model, *P* = 0.00012). We also examined common mutations between tumors and PMLs. Among significantly recurrently mutated genes in TCGA (29) and other LUAD PML studies (10, 12), *EGFR* mutations were the most common mutation in AIS/MIA and *KRAS* mutations were the most common mutation in AAH (Supplementary Fig. S5). Next, we found significant associations between histology and *EGFR* (Supplementary Table S2, χ^2^, *P* = 0.018) and *TP53* (Supplementary Table S3, χ^2^, *P* = 0.0024) driver mutations, with tumor samples more positively associated with these mutations compared with AAH and AIS/MIA samples. There was no association with driver mutation presence and histology for *KRAS* mutations (Supplementary Table S4). For patients with available exome sequencing data for both tumor and PMLs (*n* = 69 PMLs and *n* = 21 LUAD from *n* = 15 patients), we computed the Jaccard similarity based on shared deletions, insertions, and SNPs to determine relatedness between individual PMLs and their respective paired tumor. The mean similarity between tumors and PMLs from the same patient (0.0050 ± 0.017, maximum = 0.11) was significantly higher than between tumors and PMLs from unrelated patients (0.00012 ± 0.00017, maximum = 0.00063; Wilcoxon rank-sum test, *P* = 0.020). However, the similarity between PMLs and tumors from the same patient was overall low, indicating few shared somatic alterations. These results indicate that tumors have increased genomic instability compared with PMLs, as previously observed (10).

**Figure 4.**
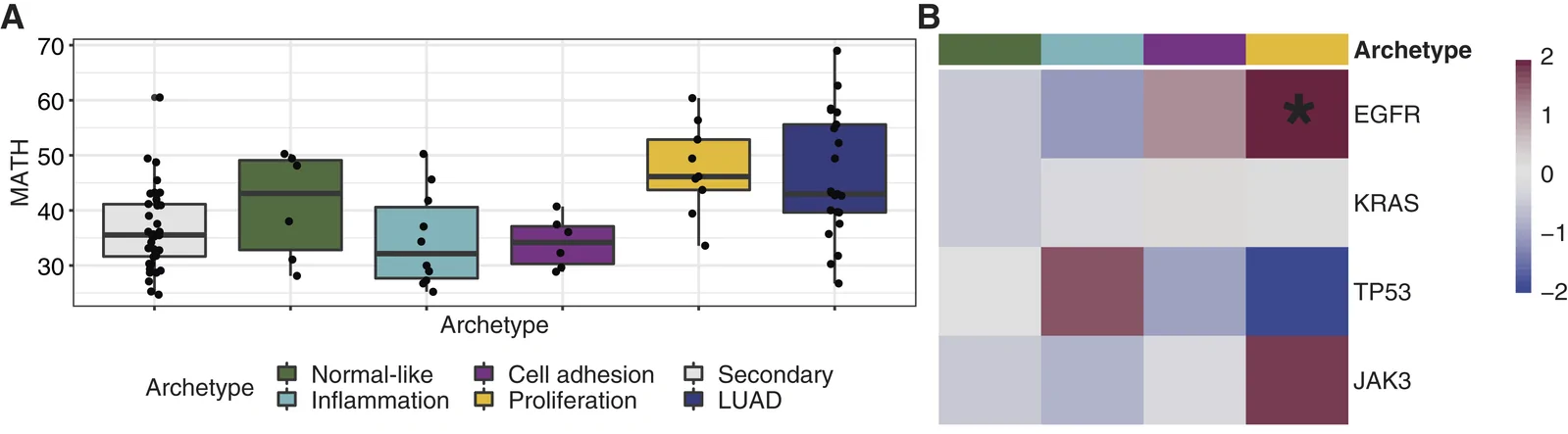
Archetypes are associated with the somatic mutation landscape. **A,** Boxplot of MATH scores for Lung PCA PML samples with archetype assignments (*n* = 6 normal-like, *n* = 10 inflammation, *n* = 6 cell adhesion, *n* = 9 proliferation, and *n* = 38 secondary) and Lung PCA tumor samples (tumor samples were not included in PML archetype analysis, *n* = 21 samples). PML samples assigned to the proliferation archetype had significantly higher MATH scores compared with all other PMLs (linear mixed-effects model with archetype vs. not as binary fixed effect and patient as a random effect, *P* < 0.0001) but were not significantly different from tumor samples (linear mixed-effects model with archetype/tumor category as a fixed effect and patient as a random effect, contrasts were used to compare individual levels). **B,** Heatmap of z-statistic for the absence/presence of each driver mutation (rows) modeled as a function of distance from archetype (columns) in a combined analysis of Lung PCA (*n* = 57 AAH and *n* = 12 AIS/MIA samples) and GSE102511 (*n* = 13 AAH samples) samples with available exome sequencing data. *EGFR* driver mutations are significantly associated with the proliferation archetype. *, *P* < 0.05 (logistic mixed-effects model with distance from each archetype and study as fixed effects and patient as a random effect).

We next assessed the relationship between archetypes and the PML somatic mutational landscape. Lung PCA PMLs assigned to the proliferation archetype had significantly increased MATH scores (Fig. 4A, linear model, *P* < 0.0001) compared with all other PML samples. When comparing the MATH scores of tumor samples with PML samples with archetype assignments, the inflammation, and cell adhesion archetypes had lower MATH scores (linear model, *P* = 0.0022 and *P* = 0.032, respectively). There was no significant difference between tumor and proliferation archetype PML sample MATH scores (*P* = 0.76), indicating a similar degree of MATH in proliferation samples and LUAD samples. The mean Jaccard similarity based on shared deletions, insertions, and SNPs for PMLs assigned to the proliferation, cell adhesion, inflammation, and normal-like archetypes and their respective paired tumor samples was 0.014 (*n* = 5 patients, maximum = 0.11), 0.00016 (*n* = 3 patients, maximum 0.0012), 0.00025 (*n* = 3 patients, maximum 0.0017), and 0.0021 (*n* = 3 patients, maximum 0.0033), respectively. Samples assigned to the proliferation archetype were the most like their paired tumor samples, but this did not meet statistical significance compared with the normal-like, inflammation, or cell adhesion archetypes (Wilcoxon rank-sum test). We also examined differences in driver mutations in a combined analysis of Lung PCA PMLs and GSE102511 samples (*n* = 13 AAH) with available targeted DNA sequencing data, focusing on mutations that appeared in at least four PMLs (Supplementary Tables S5–S8). Samples closer to the proliferation archetype were more likely to harbor *EGFR* mutations (Fig. 4B, logistic regression, *P* = 0.0021). These results suggest that PMLs have fewer somatic alterations and a weak genetic relationship with their paired tumors, although this analysis is limited by the incomplete availability of exome DNA sequencing data for all paired PML and tumor samples. However, the proliferation archetype PMLs, like tumors, were positively enriched for *EGFR* mutations and tended toward increased mutant-allele tumor heterogeneity compared with other archetypes.

### Archetypes are associated with altered immune phenotypes

**Figure 5.**
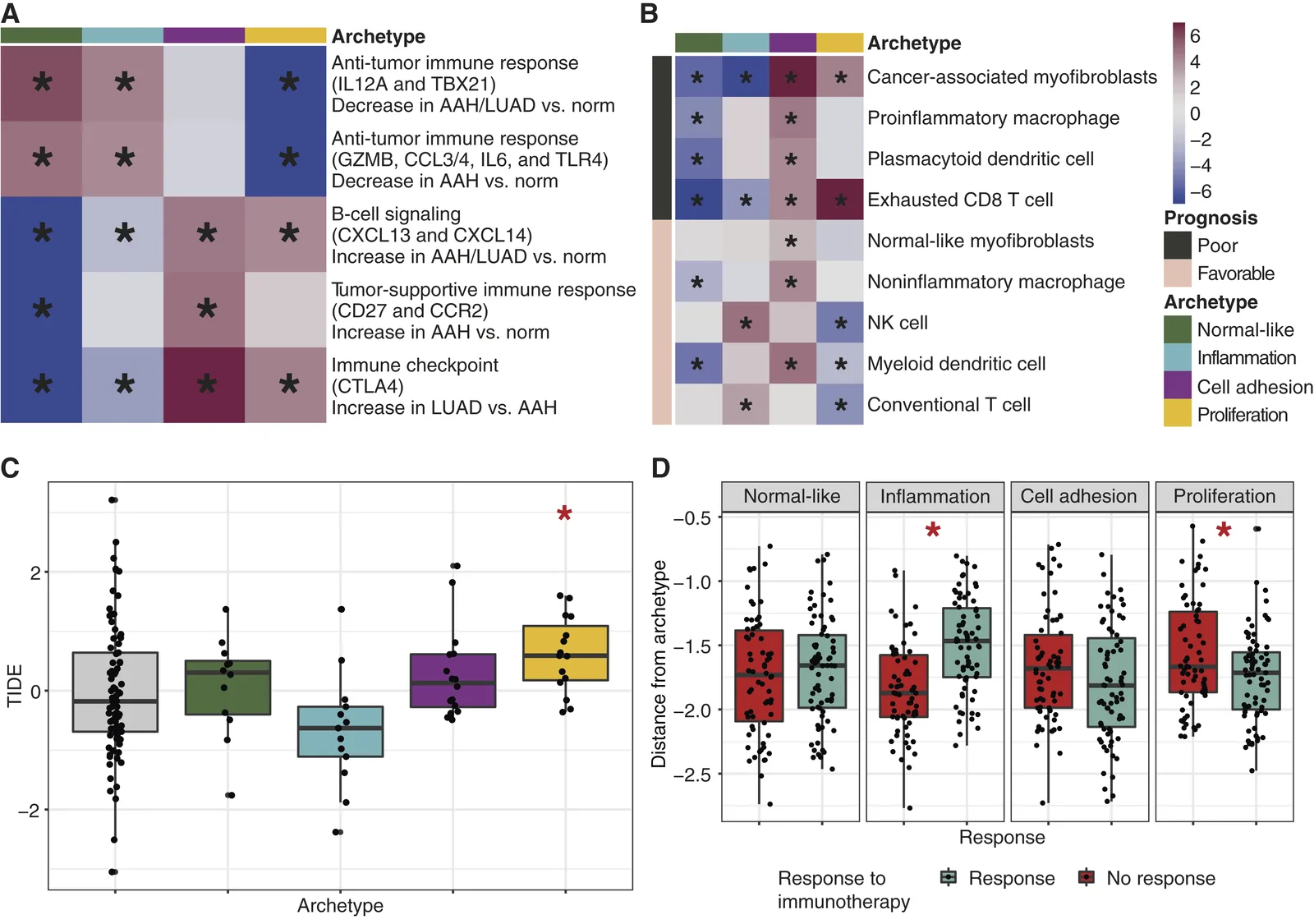
Archetypes are associated with signatures of LUAD aggression and prognosis. **A,** Heatmap of *t*-statistics for association between distance from each archetype (columns) and GSVA scores for the Sivakumar and colleagues gene signatures that differ between normal, AAH, and tumor samples (rows). *, FDR < 0.05 for the distance term (linear mixed-effects models with distance from each archetype and study as fixed effects and patient as a random effect). **B,** Heatmap of *t*-statistics for association between distance from each archetype (columns) and GSVA scores for Bischoff and colleagues cell type gene signatures (rows). *, FDR < 0.05 for the distance term (linear mixed-effects models with distance from each archetype and study as fixed effects and patient as a random effect). **C,** Boxplot of TIDE scores stratified by PML archetype assignments. Only PML samples assigned to the proliferation archetype have a significantly higher TIDE score compared with other PMLs. *, *P* < 0.05 (linear mixed-effects models with archetype vs. all other PMLs and study as fixed effects and patient as a random effect). **D,** Boxplots stratified by archetype and colored by TIDE biomarker immunotherapy response prediction. Distances closer to zero are closer to the archetype. *, significant relationship between distance from the archetype and response prediction (logistic mixed-effects model with distance from archetype and study as fixed effects and patient as a random effect, *P* < 0.05), with PMLs assigned to the inflammation archetype predicted to benefit and samples assigned to the proliferation archetype predicted to not benefit from immunotherapy.

To further understand the relationship of the archetypes to altered immune microenvironments, we used signatures from the study by Sivakumar and colleagues (GSE102511; ref. 10), which defined immune-specific gene clusters differentially expressed between normal, AAH, and LUAD samples, and cell type-specific signatures from Bischoff and colleagues (42) derived from single-cell RNA sequencing of LUAD. For each signature, we computed GSVA scores and examined whether these scores were associated with distance from each archetype (Fig. 5A and B, linear model, asterisk indicates FDR < 0.05). On both an immune pathway level (Sivakumar and colleagues, Fig. 5A) and a cell type level (Bischoff and colleagues, Fig. 5B), samples close to the cell adhesion and proliferation archetypes were significantly enriched for both protumor B-cell signaling and immune checkpoint pathways, and cancer-associated myofibroblasts and exhausted CD8^+^ T cells associated with a poor prognosis. Conversely, samples close to the normal-like and inflammation archetypes showed the opposite pattern of expression. The similarities and differences between the archetypes were also found when looking at nonimmune focused gene coexpression clusters identified by Sivakumar and colleagues (Supplementary Fig. S6A). Despite the noted similarity between the proliferation and cell adhesion archetypes, samples closer to the proliferation archetype also had a significant decrease in both antitumor immune responses and signatures for natural killer (NK) and T cells associated with better prognosis.

Using mIF performed on a subset of Lung PCA samples as described by Yanagawa and colleagues (Supplementary File S1, *n* = 39 samples total, *n* = 30 AAH and *n* = 9 AIS/MIA; ref. 47), we assessed whether the density of immune cell populations and PanCK^*+*^/Ki67^*+*^ cells varied with proximity to each archetype. Samples closer to the proliferation archetype demonstrated a decrease in Natural Killer T cells (NK T cells) (Supplementary Fig. S6B, *P* = 0.018). The added resolution of T-cell type signatures identified by Bischoff and colleagues (Fig. 5B) showed a statistically significant increase in exhausted CD8^+^ T cells and a decrease in conventional T cells, suggesting that specific CD4^+^ and CD8^+^ T-cell populations may be important to the proliferation archetype. The reverse pattern is observed for samples closer to the inflammation archetype, which have a significantly increased CD8^+^ T-cell density (*P* = 0.0066) in the mIF analysis and significantly increased NK T-cell and conventional T-cell density in the Bischoff and colleagues signatures. We also found a significant increase in density of PanCK^*+*^/Ki67^*+*^ cells in samples assigned to the proliferation archetype compared with other PMLs (Supplementary Fig. S6C, linear model, *P* = 0.037). Additionally, the density of PanCK^*+*^/Ki67^*+*^ cells is significantly correlated to GSVA scores for the cell-cycle module (Spearman rho = 0.33, *P* = 0.043) which contains *MKI67*. These results further characterize the archetype epithelial and immune microenvironments and suggest that the archetypes represent a spectrum of progressive changes to immune pathways and cell types.

Finally, we used gene markers of T-cell dysfunction and infiltration of cytotoxic T lymphocytes capable of predicting response to immune checkpoint therapy in non–small cell lung cancer (46, 56) to evaluate potential differences in the efficacy of immunotherapy (anti-PD1 and anti-CTLA4) between archetypes by calculating a TIDE score. A higher TIDE score suggests decreased efficacy of immunotherapy. We found that samples assigned to the proliferation archetype had a significantly increased TIDE score compared with all other PMLs (Fig. 5C, linear model, *P* = 0.0041). The TIDE model framework also gives a binary prediction for if a sample is likely to benefit from immunotherapy by using a combination of the TIDE score and a signature for interferon γ. As samples got closer to the proliferation archetype, they were predicted not to benefit from immunotherapy (*P* = 0.014), whereas samples closer to the inflammation archetype were predicted to benefit from immunotherapy (Fig. 5D, logistic regression, *P* = 0.00040). These results highlight a potential prognostic significance of the differing association of the archetypes with immune cell features, where the inflammation and proliferation archetypes differed in the function and density of T-cell populations.

### Archetypes are related to tumor histopathologic and clinical features in independent studies

To better understand the relationship of the archetypes to features of LUAD, including stage, histopathologic features, and prognosis, we fit data from multiple LUAD datasets to the PML polytope. We utilized transcriptional data from LUAD tumors in the Lung PCA cohort (*n* = 49 samples from *n* = 30 patients), the Yoo and colleagues study [GSE166720 (15), *n* = 33 samples, *n* = 33 patients], a prospective multicenter study that includes stages I, II, and III LUAD in the United Kingdom [TRACERx (52), *n* = 460 samples, *n* = 186 patients], TCGA (29) data that include stages I to IV (*n* = 394 samples, *n* = 394 patients), the Director’s Challenge Consortium for the Molecular Classification of stages I, II, and III LUAD [GSE68465 (48), *n* = 362 samples, *n* = 362 patients], stages I and II LUAD from studies by Okayama and colleagues and Yamauchi and colleagues [GSE31210 (49, 50), *n* = 226 samples, *n* = 226 patients], stage I LUAD from Rousseaux and colleagues [GSE30219 (51), *n* = 85 samples, *n* = 85 patients], and PMLs sampled from East Asian never-smokers by Chen and colleagues (EGAD50000000637, *n* = 25 AAH samples and *n* = 142 AIS/MIA samples, *n* = 167 patients; ref. 18). After confirming within each dataset that genes within a module were more strongly correlated with the module’s GSVA score than genes outside the module (Supplementary Fig. S7, Kolmogorov–Smirnov test, *P* < 0.0005 for all module and dataset comparisons), we fit each dataset to the PML polytope (Supplementary Fig. S8).

**Figure 6.**
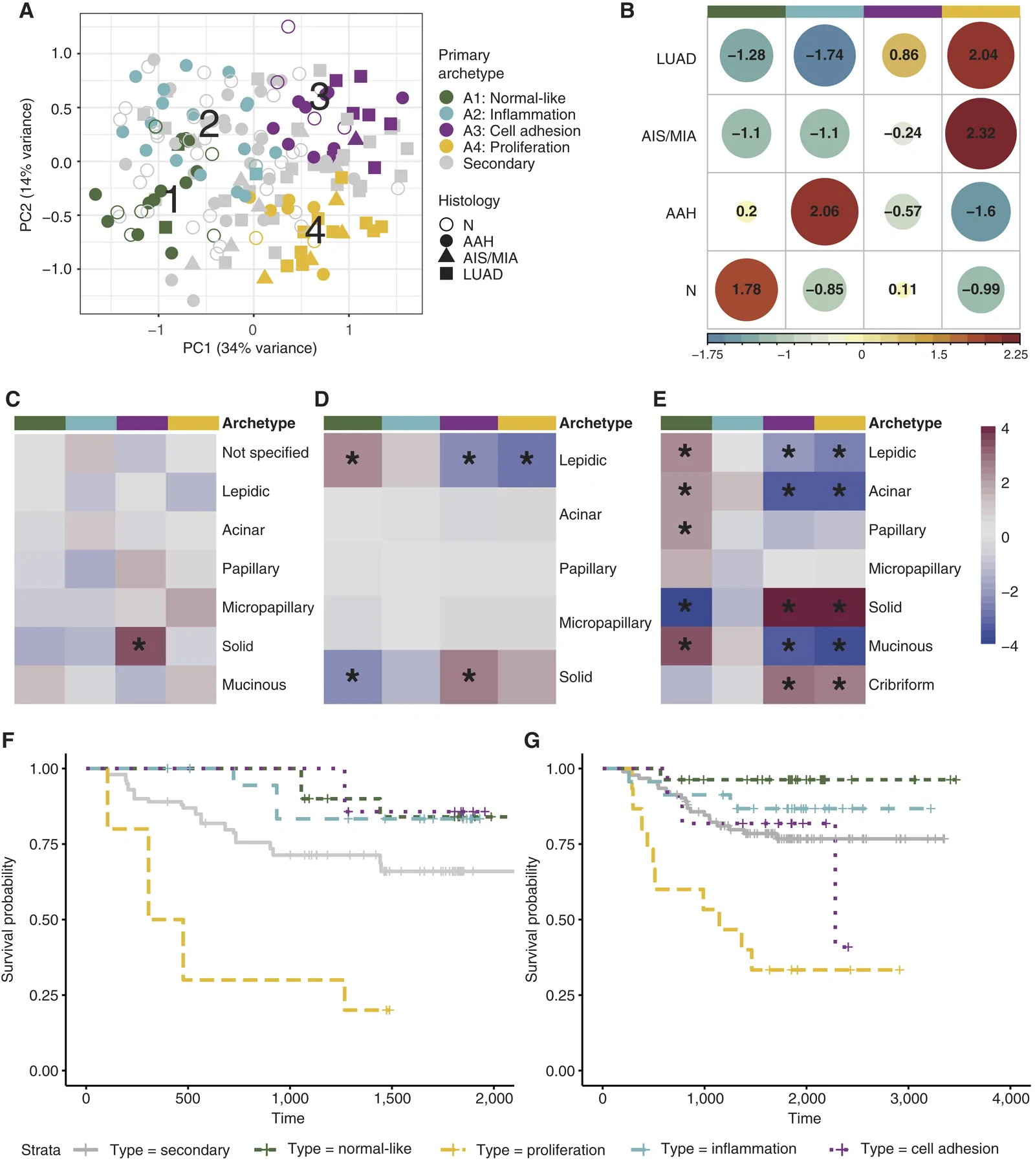
Projection of archetypes into cancer data stratifies cohorts by lesion features and prognosis. **A,** Scatterplot of first two PCs computed from GSVA scores for PML-derived modules in the full Lung PCA cohort, with annotated projection of PML archetypes into the Lung PCA PC space. Samples in the top 0.125 proportion of distances from each archetype were assigned archetypes, whereas other samples were labeled as “secondary,” with colors representing samples closest to each respective archetype. Shapes designate sample histology. **B,** Visualization of χ^2^ residuals from χ^2^ test of association of archetypes with histology in Lung PCA samples. Plot shows contribution of each cell to the test statistic, in which circle size is proportional to cell contribution. Positive (red) values specify a positive association between the corresponding row and column variables (*P* = 0.00053). **C**–**E,** Heatmap of *t*-statistics for linear models testing the association between distance from each archetype (columns) and tumor histologic pattern (rows), *, *P* < 0.05, for: **C,** Lung PCA tumor samples (linear mixed-effects models with histologic pattern and stage as fixed effects and patient as a random effect). **D,** GSE166720 tumor samples (linear models with histologic pattern as a fixed effect) and (**E**) TRACERx tumor samples, with histology indicating overall histology of tumor (linear mixed-effects models with histologic pattern and stage as fixed effects and patient as a random effect). **F** and **G,** Kaplan Meier curves stratified by archetype with Cox proportional-hazards model used to estimate disease-free survival probability for samples closest to each archetype in **F**. TRACERx stage I tumor samples (*n* = 158 samples, *n* = 77, patients, *n* = 100 secondary, *n* = 20 normal-like, *n* = 21 inflammation, *n* = 7 cell adhesion, *n* = 10 proliferation, Cox proportional-hazards model, *P* = 0.00016, HR = 4.50 for samples assigned to proliferation archetype). **G,** GSE31210 stage I tumor samples (*n* = 168 samples, *n* = 168 patients, *n* = 92 secondary, *n* = 27 normal-like, *n* = 23 inflammation, *n* = 11 cell adhesion, *n* = 15 proliferation, Cox proportional-hazards model, *P* = 0.00027, HR = 4.12 for samples assigned to proliferation archetype).

In the Lung PCA data, inclusion of the LUAD samples (*n* = 49 LUAD, *n* = 48 normal, and *n* = 80 PML) changed the archetype assignment of two PML samples (two cell adhesion archetype PMLs reassigned to “secondary”; Fig. 6A). The inclusion of LUAD samples also strengthened the association between the sample archetypes and histology (Fig. 6B; Supplementary Table S9, χ^2^, *P* = 0.00053). The proliferation archetype was enriched with LUAD and AIS/MIA samples, the inflammation archetype with AAH samples, and the normal-like archetype with normal samples. Next, we examined the archetype relationships between PMLs and their paired tumors using cosine similarity with the four distances from each archetype center for each sample. Tumors from the same patient (*n* = 21 patients with multiple tumor samples, cosine similarity, mean = 0.98 ± 0.028) have a significantly higher similarity (Wilcox test, *P* < 0.0001) compared with tumors from different patients (cosine similarity, mean = 0.96 ± 0.034). We also observed that paired PML and tumor samples (*n* = 58 PML, *n* = 26 LUAD, *n* = 21 patients, cosine similarity, mean = 0.96 ± 0.044) demonstrated increased similarity (Wilcox test, *P* = 0.0021) in the distance from archetype centers compared with PMLs and unrelated tumors (cosine similarity, mean = 0.95 ± 0.043). To understand whether archetype assignment influences tumor–PML pair similarity, we computed the cosine similarity between PMLs assigned to either the normal-like (*n* = 8 PML, *n* = 7 LUAD, *n* = 6 patients) or proliferation (*n* = 7 PML, *n* = 6 LUAD, *n* = 5 patients) archetypes with paired tumors (Supplementary Fig. S9). Only normal-like PMLs (mean = 0.92 ± 0.062), not cell adhesion or inflammation, had a significantly lower similarity than PMLs assigned to the proliferation archetype (mean = 0.97 ± 0.025) from their paired tumors (Wilcox test, *P* = 0.00017). These results indicate that paired PMLs and tumors have relatively weak similarity, similar to the findings from the exome sequencing data, and that normal-like archetype PMLs are the least similar in archetype space to their paired tumor samples.

A study by Chen and colleagues (18) sequenced PMLs obtained from East Asian never-smokers that were surgically resected based on either size (>0.8 cm), partially solid nodule density on CT, or continued growth during lesion surveillance. We projected archetypes into these samples to assess whether the relationship between histology and archetype was similar to the Lung PCA. We found that histology was significantly associated with archetype assignment, with a positive association between AAH samples and the normal-like archetype and a positive association between the separately sequenced invasive portion of MIA lesions and the proliferation archetype (Supplementary Fig. S10A, χ^2^, *P* = 0.0011). AAH samples were also significantly closer to the normal-like archetype (linear model, *P* = 0.0018), and the invasive MIA samples were significantly closer to the proliferation archetype (linear model, *P* = 0.0016). Additionally, the archetypes were associated with nodule density on CT, with the normal-like and inflammation archetypes positively associated with pure ground glass lesions, whereas the cell adhesion and proliferation archetypes were positively associated with partially solid lesions (Supplementary Fig. S10B, χ^2^, *P* = 0.056). Samples closer to the normal-like archetype were significantly associated with ground glass lesions (linear model, *P* = 0.029). These results recapitulated the association between PML histology and archetype observed in cohorts in which PMLs were primarily sampled in proximity to tumors and demonstrate an association between archetypes and CT nodule density.

Next, we examined the relationship between the PML-derived archetypes and LUAD stage, histopathologic features, and lymph node metastasis. Across multiple cohorts, samples closer to the proliferation archetype were associated with higher stage (linear model, stage II and above) and samples closer to the normal-like archetype were associated with stage I samples (linear model), with the exception of TCGA. This pattern was observed in TRACERx (*P* = 0.020 and *P* = 0.071), GSE68465 (*P* = 0.00013 and *P* = 0.0042), GSE31210 (*P* < 0.0001 and *P* < 0.0001), TCGA (*P* = 0.00057 and *P* = 0.24), and Lung PCA LUAD samples (*P* = 0.031 and *P* = 0.010), respectively. Using the tumor predominant histologic pattern (as opposed to sample-level histologic pattern), we found that samples closer to the cell adhesion archetype were enriched with the aggressive solid histologic pattern in Lung PCA (Fig. 6C, linear model, *P* = 0.0010), Yoo and colleagues (Fig. 6D, linear model, *P* = 0.010), and TRACERx LUAD samples (Fig. 6E, linear model, *P* < 0.0001). In TRACERx, samples from patients with solid predominant tumors were also significantly closer to the proliferation archetype (linear model, *P* < 0.0001). Samples from tumors with the less aggressive lepidic pattern were significantly closer to the normal-like archetype in both the Yoo and colleagues and TRACERx LUAD samples (*P* = 0.027 and *P* = 0.015, respectively). In TRACERx, the size of the invasive nonlepidic portion of the tumor was positively correlated with closeness to the proliferation archetype (Pearson R = 0.30, *P* < 0.0001). In categorically assigned GSE68465 samples (*n* = 210 samples, *n* = 210 patients), archetypes were significantly associated with tumor grade (Supplementary Fig. S11A; Supplementary Table S10; χ^2^; *P* < 0.0001) and lymph node metastasis (Supplementary Fig. S11B; Supplementary Table S11; χ^2^; *P* = 0.0038). Samples assigned to the normal-like archetype were positively associated with low-grade tumors, whereas samples assigned to the proliferation archetype were positively associated with high-grade tumors and lymph node metastasis (N0 vs. N1+). These findings were consistent when examining the relationship between sample distance from the proliferation archetype and tumor grade or lymph node metastasis (linear model) in the full GSE68465 dataset (*P* = 0.0039 and *P* = 0.0036, respectively), Lung PCA tumor samples (*P* = 0.0051 and *P* = 0.081), TCGA (tumor grade not available, *P* = 0.00030 for lymph node metastasis), and TRACERx (tumor grade not available, *P* = 0.031 for lymph node metastasis). In the TRACERx dataset in which the presence of lymphovascular invasion was annotated, samples closer to the normal-like archetype were associated with the absence of lymphovascular invasion, whereas the opposite was true for the proliferation archetype (linear model, *P* = 0.020 and *P* = 0.021, respectively). Taken together, the proliferation archetype was associated with the aggressive solid predominant histologic pattern, tumor invasive size, high tumor grade, lymph node metastasis, and lymphovascular invasion, suggesting that this archetype is associated with aggressive LUAD compared with the other archetypes.

Given the association between the proliferation archetype and aggressive LUAD features, we wanted to determine whether the proliferation archetype was associated with poor lung cancer–specific disease-free survival. As stage I LUADs are likely to be treated with surgical resection without additional therapy, we chose to focus the analysis on this subset of LUAD. In both the TRACERx stage I data (*n* = 158 samples, *n* = 77 patients, Fig. 6F) and GSE31210 stage I data (*n* = 168 samples, *n* = 168 patients, Fig. 6G), there was a significant difference in disease-free survival time between samples assigned to the different archetypes, with shorter disease-free survival in samples assigned to the proliferation archetype (*P* = 0.00016, HR = 4.50 and *P* = 0.00027, HR = 4.12, respectively). Using sample distance to the proliferation archetype, we found that stage I TRACERx LUAD samples closer to the proliferation archetype trended toward shorter disease-free survival (*P* = 0.14, HR = 3.31). Stage I LUAD samples from GSE31210 and GSE30219 closer to the proliferation archetype were also significantly associated with worse prognosis (Supplementary Fig. S12, *P* = 0.00086, HR = 3.31 and *P* = 0.0088, HR = 3.08, respectively). These results suggest that the proliferation archetype is associated with worse disease-free survival in stage I LUAD samples in multiple independent datasets.

## Discussion

Wider adoption of lung cancer screening with low-dose CT has increased the detection of pure GGOs that can represent premalignant histology (57), presenting a need to develop biomarkers that can distinguish between indolent and aggressive PMLs that will progress to invasive lung cancer (6). Prior work profiling LUAD PMLs using bulk RNA and exome sequencing focused on identifying similarities and differences between PMLs and tumors (8, 10–12, 14, 15, 17). However, a recent study of East Asian never-smokers found few differentially expressed genes by pathologic stage but more changes associated with radiologic features (18). There is an unmet need to characterize molecular heterogeneity in LUAD PMLs and find subtypes associated with prognosis (58, 59) to develop biomarkers to prevent overtreatment of indolent PMLs and hasten treatment of aggressive PMLs, such as those detected via current lung cancer screening programs (7, 60). To this end, using LUAD PMLs profiled by RNA-seq from four cohorts, we identified nine conserved gene coexpression modules. Of these modules, four had significantly altered expression between pathologic stage (AAH compared with AIS/MIA) in the Lung PCA cohort. These nine modules were subsequently used to define four archetypes—normal-like, inflammation, cell adhesion, and proliferation—that represent different gene expression states among PMLs and are associated with activation of genes involved in distinct biological pathways, immune microenvironments, and somatic mutation profiles. The gene expression patterns that define each archetype were able to discriminate between aggressive and indolent LUAD across multiple studies, including CT nodule density of PMLs, linking PML molecular heterogeneity and prognostic histopathologic features in LUAD.

PMLs representative of the proliferation archetype were associated with the presence of *EGFR* driver mutations, increased mutational heterogeneity, an altered immune landscape, and increased expression of cell proliferation and metabolic pathways, suggestive of rapid cell growth. Previously, the frequency of *EGFR* mutations had been found to increase as lesions progress along the histologic spectrum from AAH to AIS/MIA to LUAD (18), and *EGFR* mutations were more frequent in tumors compared with AAH (10). Our study similarly found that *EGFR* mutations were enriched in tumor samples compared with PMLs. However, within PMLs, we found that the proliferation archetype (enriched for AIS/MIA) was enriched for *EGFR* mutation compared with PMLs from all other archetypes. We also demonstrated that proliferation archetype PMLs have the highest levels of intralesion mutational heterogeneity, similar to tumor samples. The proliferation archetype was also associated with both tumor-supportive immune pathways identified by Sivakumar and colleagues (10) and signatures for proinflammatory macrophages and exhausted CD8^+^ T-cell populations identified by Bischoff and colleagues as being associated with a poor prognosis (42). A spatial analysis of AAH, AIS/MIA, and LUAD similarly found that regions with decreased innate inflammatory signaling and increased immune evasion were associated with increases in PDL1^*+*^Ki67^*+*^ epithelial cells (61), consistent with the cell cycle-related gene expression enriched in the proliferation archetype. Interestingly, tumors most similar to the proliferation archetype had significantly decreased disease-free survival time, were enriched for aggressive histologic patterns and invasive features, and had higher grade compared with tumors most similar to the other archetypes. Collectively, these findings indicate that some PMLs share gene expression patterns with aggressive LUAD. This further suggests that patients with PMLs similar to the proliferation archetype may benefit from more frequent CT screening, targeted interception strategies, or resection.

In contrast to the proliferation archetype, PMLs representative of the normal-like archetype shared module gene expression patterns with histologically normal tissues adjacent to tumors, or with lepidic tumors in studies of LUAD, and lacked significant associations with known driver mutations. Normal-like archetype samples had increased expression of genes involved in pathways regulating endothelial cell proliferation, potentially reflecting the critical role endothelial cells have in alveolar gas exchange in normal lung tissue (61). Additionally, normal-like archetype samples showed increased expression of genes in antitumor immune pathways (10) and decreased expression of tumor-associated cell type signatures such as cancer-associated myofibroblasts, proinflammatory macrophages, and exhausted CD8^+^ T cells (42). This potentially represents a subset of lesions similar to those observed by Krysan and colleagues (12) that exhibit active immune surveillance. Normal-like archetype PMLs may represent a subset of lesions that are unlikely to progress to cancer (7) and could be managed conservatively via repeat CT imaging.

The inflammation and cell adhesion archetypes share module gene expression with both the normal-like and proliferation archetypes and potentially represent intermediate LUAD PML phenotypes. The inflammation archetype, similar to the normal-like archetype, demonstrated elevated expression of antitumor immune pathways (10) and cell type–specific markers of NKT and conventional T cells that were associated with a more favorable prognosis (42). Collectively, these data suggest that inflammatory archetype PMLs may be recognized by the immune system (10–12, 14, 15, 47). Samples representative of the cell adhesion archetype shared some features with the proliferation archetype, including increased expression of immune checkpoint pathways (10) and signatures for cancer-associated myofibroblasts and exhausted CD8^+^ T cells associated with a less favorable prognosis (42). In contrast with proliferation archetype samples, lesions close to the inflammation and cell adhesion archetypes had significantly lower intralesion mutational heterogeneity compared with tumors. Unlike the proliferation archetype, LUAD tumors closest to the cell adhesion archetype were not associated with decreased disease-free survival, higher stage, higher grade, or lymphovascular invasion. The cell adhesion archetype samples displayed increased expression of the PML-derived extracellular matrix module, the Hallmark epithelial-to-mesenchymal transition pathway, and higher expression of a cancer-associated myofibroblast gene signature (42). These findings not only suggest that PMLs vary with respect to their level of stromal interaction but also raise the question about the role of these stromal interactions in early tumorigenesis, as recently highlighted in studies of AIS/MIA (17, 18). Our characterization suggests the archetypes may represent a spectrum of indolent to aggressive transcriptional patterns among LUAD PMLs, with the cell adhesion archetype representing an intermediate between the inflammation and proliferation archetypes. The alterations in the stromal and immune features in the cell adhesion archetype compared with the proliferation archetype may modulate unchecked proliferation, leading to a less aggressive phenotype.

To determine whether the transcriptional patterns that define the PML archetypes are associated with progression to lung cancer, future multi-omics studies are needed to molecularly, radiologically, and pathologically phenotype PMLs over time. Like many published works characterizing LUAD PMLs, the lesions profiled in this study were identified within lung resections taken from patients with LUAD and may be affected by field cancerization (53). We are also unable to fully understand how these PMLs may evolve over time as they are removed at resection. The reproducible association of the normal-like and proliferation archetypes with less and more aggressive PML features, respectively, in PMLs obtained in the absence of LUAD tumors profiled by Chen and colleagues, suggests that these archetypes may help identify aggressive PMLs; however, validation in larger and more diverse cohorts is needed. LUAD PMLs are small and located deep in lung parenchyma, making them inaccessible to biopsy by standard bronchoscopy, which complicates longitudinal sampling (59). As techniques such as robotic-assisted (62) and electromagnetic navigation bronchoscopy (63) become more widely available and accurate, and evidence continues to build about the diverse natural history of LUAD PMLs (7, 57, 58), sampling of PMLs, pure GGOs, and part-solid GGOs may become more routine both clinically and to study disease. Future work analyzing spatial transcriptomic profiling of PMLs is needed to identify cell types and immune microenvironments associated with the archetypes and further explore the influence of adjacent tumors.

In summary, our archetyping approach identified PML-variable features across multiple datasets to characterize PML heterogeneity. These features associate with pathway dysregulation and LUAD tumor stage, histopathologic features, and prognosis. Molecular signatures that can distinguish between indolent and aggressive PMLs may help personalize lung cancer screening protocols, prevent overdiagnosis, and allow for treatment that intercepts the development of aggressive LUAD via surgery and/or therapy to prevent cancer progression. Future longitudinal studies that monitor and sample nodules over time will be necessary to determine the direct relationship between PML archetypes, progression to lung cancer, and the histopathologic and prognostic features of the LUAD that may develop from a PML.

## Supplementary Material

Supplementary File 1 Lung PCA sample information.

Supplementary File 2 Enrichment of GO Biological Process terms in PML-derived gene modules.

Supplementary File 3 Genes in each PML-derived module.

Supplementary Figure 1 PML-derived module co-expression (A) Density plot of correlation of each gene’s expression values to module GSVA score stratified by genes within the module and genes outside the module for all PML data (left) and PML data from each cohort (B-D) Boxplots of GSVA scores computed for each PML-derived module across (B) GSE102511 samples (*n* = 47 samples) stratified by module and colored by histology. Asterisk (*) indicates histology is significantly different from AAH samples (linear mixed effects model with histology as fixed effect and patient as random effect, p < 0.05) (C) GSE193725 samples (*n* = 51 samples) stratified by module and colored by histology. Asterisk indicates histology is significantly different from AIS/MIA samples (linear mixed effects model with histology as fixed effect and patient as random effect, p < 0.05) (D) GSE166720 samples (*n* = 50 samples) stratified by module and colored by histology. Asterisk indicates histology is significantly different from AIS/MIA samples (linear model, p < 0.05).

Supplementary Figure 2 Optimization of archetype analysis (A) Line graph of explained variance versus number of potential archetypes (k = 2 to k = 10), with red dashed line showing suggested number of archetypes based on elbow. Elbow computed by finding the most distant point from the line that passes through k = 2 to kmax = 10 (B) Line graph of t-ratio versus number of potential archetypes (k = 3 to k = 6) to confirm that k = 4 is the optimal number of archetypes. We computed empirical p-values by using randomized gene modules to calculate GSVA scores. We performed this randomization 5000 times and recomputed the t-ratio for k = 3, k = 4, k = 5, and k = 6 for each iteration. Using these t-ratios as a test statistic, we determined how often the t-ratio in the randomized data was greater than the t-ratio of the PML data, and the p-value from the permutation test for each value of k is annotated, with a red dashed line showing the first value for k with a significant p-value at k = 4.

Supplementary Figure 3 GSEA of Hallmark gene sets among genes associated with distance from archetype Bar plot stratified by archetype and categorized by pathway type to show significant positive associations (FDR<0.05) of mSigDB “Hallmark” gene sets with distance from archetypes using Gene Set Enrichment Analysis. Using limma with a model that included distance from the archetype, study, and RNA quality (Methods) as fixed effects and patient as a random effect, genes were ranked by t-statistic for their association with distance from each archetype. Bars are colored based on value of normalized enrichment score for each gene set, and only sets with a positive normalized enrichment score and FDR<0.05 are shown.

Supplementary Figure 4 Additional characterization of PML archetypes (A-C) Visualization of χ^2^ residuals from χ^2^ test of association of archetypes with: (A) study (*n* = 17 GSE102511, *n* = 17 GSE166720, *n* = 23 GSE193725, *n* = 80 Lung PCA PML). No archetype is significantly associated with study (p = 0.33) (B) smoking status (*n* = 17 never, *n* = 104 ever, *n* = 17 unknown). No archetype is significantly associated with smoking status (p = 0.29) (C) sex (*n* = 105 female, *n* = 33 male). No archetype is significantly associated with sex (p = 0.65).

Supplementary Figure 5 Shared mutations across histology and archetypes (A) Plot visualizing frequency of single nucleotide mutations in Lung PCA samples. The top bar graph shows the frequency of mutations for each sample. The right bar graph shows the frequency of mutations in each gene Each column represents a sample, and each row corresponds to a different gene previously identified as a cancer driver in studies of LUAD and LUAD PMLs. Sample histology and archetype are annotated in bottom 2 rows. Samples without mutations in these genes (*n* = 16 samples) not shown.

Supplementary Figure 6 Characterization of PML archetypes using AAH-related gene signatures and mIF (A) Heatmap of t-statistics for association between distance from each archetype (columns) and GSVA scores for each Sivakumar and colleagues histology-associated gene signature (rows). Asterisk (*) indicates FDR<0.05 for the distance term (linear mixed effects models with distance from each archetype and study as a fixed effects and patient as a random effect) (B) Heatmap of t-statistics from linear mixed effects model showing associations between cell type density among Lung PCA PML samples profiled with mIF and distance from each archetype (asterisk indicates p < 0.05, linear mixed effects model with distance as fixed effect and patient as random effect) (C) Boxplot of PanCK+/Ki67+ cell density per mm2 (x103) among samples with assigned archetype (asterisk indicates p < 0.05, linear mixed effects model with archetype assignment as fixed effect and patient as random effect).

Supplementary Figure 7 Correlation of gene expression to PML-derived module GSVA scores across LUAD datasets Density plot of correlation of each gene’s expression to module GSVA score colored by correlation of genes within the module (pink) and genes outside the module (purple) across the following datasets (left to right): Lung PCA samples, Lung PCA tumor samples, GSE166720 tumor samples, Chen and colleagues PML samples, and independent LUAD tumor data sets.

Supplementary Figure 8 PML archetypes in LUAD cohorts (A-H) Scatterplot of first two principal components computed from GSVA scores for PML-derived modules in LUAD datasets, with annotated projection of PML archetypes (A1-A4) into principal component space. Samples in the top 0.125 proportion of distances from each archetype were assigned archetypes, while other samples were labeled as “secondary”, with colors representing samples in the bin closest to archetype (A) Lung PCA tumor samples (B) Yoo and colleagues tumor samples (C) TRACERx tumor samples (D) GSE68465 tumor samples (E) GSE31210 tumor samples (F) GSE30219 tumor samples (G) TCGA tumor samples (H) Chen and colleagues PML samples.

Supplementary Figure 9 Distribution of archetype assigned samples across Lung PCA patients Scatterplot of first two principal components computed from GSVA scores for PML-derived modules in the Lung PCA cohort. The plots are stratified by patient and samples are annotated based on their assigned archetype. Samples in the top 0.125 proportion of distances from each archetype were assigned archetypes (as shown in Figure 6A), while other samples were labeled as “secondary”, with colors representing each unique archetype. Shapes designate sample histology.

Supplementary Figure 10 Association of archetypes with histology and nodule density in an independent cohort of PML Visualization of χ^2^ residuals from χ^2^ test of association of archetypes with histology and nodule density in Chen and colleagues PML data. Plot shows contribution of each cell to the test statistic, where circle size is proportional to cell contribution. Positive (red) values specify a positive association between the corresponding row and column variables (A) Histology, where the authors sequenced the invasive portion of MIA lesions separately from the in situ parts of the lesion (p = 0.0011) (B) Nodule density (p = 0.056).

Supplementary Figure 11 Association of archetypes with LUAD features in GSE68465 Visualization of χ^2^ residuals from χ^2^ test of association of archetypes with tumor features in GSE68465 study. Plot shows contribution of each cell to the test statistic, where circle size is proportional to cell contribution. Positive (red) values specify a positive association between the corresponding row and column variables (A) Tumor grade in GSE68465 cohort (p < 0.0001) (B) Lymph node invasion (*N*0 versus *N*1 or above) in GSE68465 cohort (p = 0.0038).

Supplementary Figure 12 Association of distance from proliferation archetype with lung cancer-specific disease-free survival in stage I LUAD data For Stage I tumors in each dataset (y-axis), the hazard ratio (x-axis) was estimated using a Cox proportional-hazards model of disease-free survival as a function of proximity to the proliferation archetype. The upper and lower 95% confidence intervals are shown as whiskers from the hazard ratio. The data sets with survival data were: TRACERx (*n* = 158 samples from *n* = 77, patients, p = 0.14, Hazard Ratio = 3.32), TCGA (*n* = 108 samples from *n* = 108 patients, p = 0.30, Hazard Ratio = 1.43), GSE68465 (*n* = 223 samples from *n* = 223 patients, p = 0.60, Hazard Ratio = 1.11), GSE31210 (*n* = 168 samples from *n* = 168 patients, p = 0.00086, Hazard Ratio = 3.31), and GSE30219 (*n* = 85 samples from *n* = 85 patients, p = 0.0088, Hazard Ratio = 3.08).

Supplementary Table 1 PML histology groups stratified by archetype across all PML data.

Supplementary Table 2 Presence of EGFR driver mutations stratified by histology in Lung PCA data.

Supplementary Table 3 Presence of TP53 driver mutations stratified by histology in Lung PCA data.

Supplementary Table 4 Presence of KRAS driver mutations stratified by histology in Lung PCA data.

Supplementary Table 5 Presence of EGFR driver mutations in PML stratified by dataset.

Supplementary Table 6 Presence of TP53 driver mutations in PML stratified by dataset.

Supplementary Table 7 Presence of KRAS driver mutations in PML stratified by dataset.

Supplementary Table 8 Presence of JAK3 driver mutations in PML stratified by dataset.

Supplementary Table 9 Lung PCA histology groups stratified by archetype.

Supplementary Table 10 Tumor grade stratified by archetype assignment in GSE68465 cohort.

Supplementary Table 11 Lymph node involvement stratified by archetype assignment in GSE68465 cohort.

## Data Availability

Bulk RNA and DNA exome sequencing data generated in this study are publicly available in GEO under accession number GSE319666 and in the Human Tumor Atlas Network Data Portal using the identifications provided in Supplementary File 1. Access to the raw sequencing data requires Database of Genotypes and Phenotypes (dbGaP) approval, which can be requested at the dbGaP study page under the HTAN study phs002371. Additional bulk RNA-seq and microarray data analyzed in this study are publicly available from GEO under accession numbers GSE102511, GSE166720, GSE193725, GSE68465, GSE31210, and GSE30219 and from the European Genome-phenome Archive under accession EGAD50000000637. TCGA RNA-seq data are available on the National Cancer Institute Genomic Data Commons Data Portal under project TCGA-LUAD. TRACERx RNA-seq data are available on Zenodo under records 7683605 and 7603386. Hallmark and Gene Ontology gene sets were acquired from MSigDB (35, 43). All other raw data generated in this study are available upon request to the corresponding author. All code is available via the GitHub repository: https://github.com/kelley27/PML_Archetypes.
