# Supplementary material for: Archetype Analysis of Lung Adenocarcinoma Premalignancy Links Heterogeneity in Premalignant Lesions to Diverging Features of Invasive Disease
Source: Mol Cancer Res. 2026 May 6;24(9):737–53. doi: 10.1158/1541-7786.MCR-25-1093 (PMC13455826; doi:10.1158/1541-7786.MCR-25-1093)
Supplement: Supplementary file 1 [file mcr-25-1093_supplementary_table_10_suppst10.docx]

**Supplementary Table 10: Tumor grade stratified by archetype assignment in GSE68465 cohort**

|  | Low Grade | Intermediate Grade | High Grade |
| --- | --- | --- | --- |
| Normal-like | 20 | 26 | 3 |
| Inflammation | 9 | 17 | 20 |
| Cell Adhesion | 1 | 29 | 26 |
| Proliferation | 2 | 22 | 35 |
