# Supplementary material for: Archetype Analysis of Lung Adenocarcinoma Premalignancy Links Heterogeneity in Premalignant Lesions to Diverging Features of Invasive Disease
Source: Mol Cancer Res. 2026 May 6;24(9):737–53. doi: 10.1158/1541-7786.MCR-25-1093 (PMC13455826; doi:10.1158/1541-7786.MCR-25-1093)
Supplement: Supplementary file 2 [file mcr-25-1093_supplementary_table_11_suppst11.docx]

**Supplementary Table 11: Lymph node involvement stratified by archetype assignment in GSE68465 cohort**

|  | N0 | N1+ |
| --- | --- | --- |
| Normal-like | 39 | 10 |
| Inflammation | 37 | 9 |
| Cell Adhesion | 35 | 21 |
| Proliferation | 31 | 28 |
