# Supplementary material for: Archetype Analysis of Lung Adenocarcinoma Premalignancy Links Heterogeneity in Premalignant Lesions to Diverging Features of Invasive Disease
Source: Mol Cancer Res. 2026 May 6;24(9):737–53. doi: 10.1158/1541-7786.MCR-25-1093 (PMC13455826; doi:10.1158/1541-7786.MCR-25-1093)
Supplement: Supplementary file 3 [file mcr-25-1093_supplementary_table_1_suppst1.docx]

**Supplementary Table 1: PML histology groups stratified by archetype across all PML data**

|  | AAH | AIS/MIA |
| --- | --- | --- |
| Normal-like | 10 | 2 |
| Inflammation | 10 | 3 |
| Cell Adhesion | 10 | 6 |
| Proliferation | 7 | 8 |
| Secondary | 48 | 33 |
