# Supplementary material for: Archetype Analysis of Lung Adenocarcinoma Premalignancy Links Heterogeneity in Premalignant Lesions to Diverging Features of Invasive Disease
Source: Mol Cancer Res. 2026 May 6;24(9):737–53. doi: 10.1158/1541-7786.MCR-25-1093 (PMC13455826; doi:10.1158/1541-7786.MCR-25-1093)
Supplement: Supplementary file 5 [file mcr-25-1093_supplementary_table_3_suppst3.docx]

**Supplementary Table 3: Presence of TP53 driver mutations stratified by histology in Lung PCA data**

|  | Present | Absent |
| --- | --- | --- |
| AAH | 3 | 49 |
| AIS/MIA | 1 | 11 |
| ADC | 9 | 17 |
