# Supplementary material for: Archetype Analysis of Lung Adenocarcinoma Premalignancy Links Heterogeneity in Premalignant Lesions to Diverging Features of Invasive Disease
Source: Mol Cancer Res. 2026 May 6;24(9):737–53. doi: 10.1158/1541-7786.MCR-25-1093 (PMC13455826; doi:10.1158/1541-7786.MCR-25-1093)
Supplement: Supplementary file 6 [file mcr-25-1093_supplementary_table_4_suppst4.docx]

**Supplementary Table 4: Presence of KRAS driver mutations stratified by histology in Lung PCA data**

|  | Present | Absent |
| --- | --- | --- |
| AAH | 8 | 44 |
| AIS/MIA | 2 | 10 |
| ADC | 4 | 22 |
