# Supplementary material for: Archetype Analysis of Lung Adenocarcinoma Premalignancy Links Heterogeneity in Premalignant Lesions to Diverging Features of Invasive Disease
Source: Mol Cancer Res. 2026 May 6;24(9):737–53. doi: 10.1158/1541-7786.MCR-25-1093 (PMC13455826; doi:10.1158/1541-7786.MCR-25-1093)
Supplement: Supplementary file 10 [file mcr-25-1093_supplementary_table_8_suppst8.docx]

**Supplementary Table 8: Presence of JAK3 driver mutations in PML stratified by dataset**

|  | Present | Absent |
| --- | --- | --- |
| GSE102511 | 2 | 11 |
| Lung PCA | 3 | 61 |
