# Supplementary material for: Archetype Analysis of Lung Adenocarcinoma Premalignancy Links Heterogeneity in Premalignant Lesions to Diverging Features of Invasive Disease
Source: Mol Cancer Res. 2026 May 6;24(9):737–53. doi: 10.1158/1541-7786.MCR-25-1093 (PMC13455826; doi:10.1158/1541-7786.MCR-25-1093)
Supplement: Supplementary file 11 [file mcr-25-1093_supplementary_table_9_suppst9.docx]

**Supplementary Table 9: Lung PCA histology groups stratified by archetype**

|  | Normal | AAH | AIS/MIA | LUAD |
| --- | --- | --- | --- | --- |
| Normal-like | 7 | 11 | 0 | 2 |
| Inflammation | 2 | 17 | 0 | 1 |
| Cell Adhesion | 4 | 9 | 1 | 7 |
| Proliferation | 2 | 6 | 4 | 10 |
| Secondary | 33 | 25 | 7 | 29 |
